# The Relevance of Insomnia Among Healthcare Workers: A Post-Pandemic COVID-19 Analysis

**DOI:** 10.3390/jcm14051663

**Published:** 2025-02-28

**Authors:** Carlos Roncero, José Bravo-Grande, Diego Remón-Gallo, Pilar Andrés-Olivera, Candela Payo-Rodríguez, Alicia Fernández-Parra, Lourdes Aguilar, Marta Peña, Armando González-Sánchez

**Affiliations:** 1 Health Science Faculty, Miguel de Cervantes European University (UEMC), Valladolid (Spain). C. del Padre Julio Chevalier, 2, 47012 Valladolid, Spain; drcarlosroncero@gmail.com (C.R.); afernandezp@uemc.es (A.F.-P.);; 2 Instituto de Investigación Biomédica de Salamanca (IBSAL), Hospital Virgen de la Vega, 10 ª Planta, Paseo de San Vicente, 58-182, 37007 Salamanca, Spain; jlbravo@saludcastillayleon.es (J.B.-G.); d.remon@usal.es (D.R.-G.); mpolivera@saludcastillayleon.es (P.A.-O.); lourdesaguilar@usal.es (L.A.); 3 Psychiatric Unit, School of Medicine, University of Salamanca (Spain), Campus Miguel de Unamuno, Calle Alfonso X El Sabio s/n, 37007 Salamanca, Spain; 4 Network of Research in Primary Care of Addictions (RIAPAD), Instituto Carlos III (Spain), 28029 Madrid, Spain; 5 Department of Occupational Health—Prevention of Occupational Risks, Salamanca Health Area & University of Salamanca Health Care Complex, Paseo de San Vicente 58-182, 37007 Salamanca, Spain; 6 Psychiatry Service, University of Salamanca Health Care Complex, Paseo de San Vicente 58-182, 37007 Salamanca, Spain; cpayo@saludcastillayleon.es; 7 Facultad de Psicología, Universidad Pontificia de Salamanca (UPSA), C/Compañía, 5, 37002 Salamanca, Spain

**Keywords:** insomnia, mental health impact, shift work, occupational hazard, anxiety, depression, COVID-19 sequelae, multidisciplinary intervention, quality of care, sleep disorders

## Abstract

**Background:** Insomnia significantly impairs healthcare worker (HCW) well-being, particularly amid COVID-19 sequelae and shift work demands. We aimed to assess the prevalence of insomnia among HCWs, identify those needing clinical intervention, analyze shift work as a potential risk factor, and explore associations with COVID-19 sequelae and psychiatric comorbidities. **Methods:** A cross-sectional online survey was administered at the University of Salamanca University Care Complex (CAUSA) from March 2023 to January 2024. Validated scales (Insomnia Severity Index, Patient Health Questionnaire-4, Generalized Anxiety Disorder Scale-2) were used to measure insomnia, depression, and anxiety. Participants scoring ISI ≥ 7 were invited for Occupational Medicine follow-up. Descriptive and inferential analyses were performed. **Results:** Overall, 1121 HCWs participated (mean age 44.59 ± 11.78, 78.3% women). The mean ISI score was 10.5 ± 5.8 (subclinical insomnia), with 22.7% reporting moderate and 3% reporting severe insomnia. Depression and anxiety affected 28.4% and 33% of respondents, respectively. Shift workers had poorer sleep (mean ISI 11.3 ± 0.9 vs. 8.8 ± 0.3, *p* < 0.001). Individuals reporting COVID-19 sequelae were 3.1 times more likely to have insomnia than those who did not (mean ISI 13.89 ± 5.9 vs. 10.33 ± 5.7, *p* < 0.001). Over one-quarter reported at least the monthly use of sleep or psychiatric medications. **Conclusions:** Insomnia remains prevalent among HCWs, influenced by shift work, COVID-19 sequelae, and mental health factors. Targeted, multidisciplinary interventions, e.g., workplace policy changes, mental health programs, and shift schedule adjustments are urgently needed to safeguard well-being, reduce burnout, and maintain quality patient care. Ensuring adequate sleep is central to minimizing errors and preserving professional performance. Future studies should investigate the impact of coordinated workplace strategies to effectively address insomnia.

## 1. Introduction

Sleep disturbances such as insomnia, sleep apnea, and circadian rhythm disorders are considered a public health epidemic [1]. They are common in healthcare workers (HCWs), who frequently face clinical situations with a high emotional burden and can perpetuate the high rates of associated burnout [2]. Insomnia can lead to significant morbidity and exacerbate medical and psychiatric conditions. Moreover, poor sleep, whether in quantity or quality, increases the risk of premature aging, obesity, and cardiovascular diseases [3,4].

Insomnia comprises daytime and nighttime symptoms that seriously affect quality of life and well-being. It is characterized by difficulty falling asleep or by fatigue or dysfunction during the day [4,5]. HCWs often exhibit a high prevalence of insomnia, with aggravating factors such as shift work [6]. In addition, the coexistence of insomnia and mental illness is common. In a systematic review and meta-analysis on the prevalence of depression, anxiety, and insomnia among HCWs during the COVID-19 pandemic (n = 33,062) [7], the prevalence of insomnia was estimated at 38.9%. The authors also found a pooled prevalence of anxiety and depression of 23.2% and 22.8%, respectively. As shown by studies performed on HCWs worldwide, including Spain, this scenario was aggravated after the pandemic [8,9,10,11,12,13,14,15,16] and requires specific measures to prevent it from becoming chronic.

This study is part of a more ambitious study. Because worrying levels of sleep problems were detected among the working population of the University of Salamanca Health Care Complex (known by its Spanish acronym CAUSA), it was decided to extend this screening to the whole workforce of the hospital. So, the aim was to first detect the workers affected by sleep problems and who wanted specific treatment. Subsequently, and with the people detected, these data would be provided to the occupational medicine unit, so that they could offer them group treatment and continue to evaluate them. Occupational medicine would then refer the HCWs who were particularly affected and who wanted it to the mental health unit for specialized treatment using both psychological and pharmacological therapy.

In view of these data, the Services of Psychiatry and Occupational Health of CAUSA carried out a study on the sleep disturbances experienced by the HCWs of this center after the COVID-19 pandemic. The objectives were as follows: (1) to evaluate the prevalence of insomnia among HCWs; (2) to identify individuals with insomnia who could be referred to the Occupational Medicine Service to undergo interventions to improve sleep quality; (3) to characterize the job profiles most affected by insomnia; (4) to analyze the relationship between sleep problems and the consequences of COVID-19; (5) to evaluate psychiatric comorbidities and their pharmacological treatment; and (6) to evaluate the presence of depression and anxiety.

## 2. Materials and Methods

A cross-sectional study was carried out through a survey completed by HCWs of CAUSA. An online questionnaire was designed (see Appendix S1) and made available via the CAUSA intranet. The HCWs were invited to participate in the study through posters on the walls (see the Supplementary Files) and advertisements on the institution’s website, and emails were sent to all HCWs from Human Resources, inviting them to participate in the survey. Participants completed the questionnaire between 7 March 2023 and 5 January 2024.

The questionnaire consisted of 70 questions grouped into 8 blocks and had an estimated response time of 15 min. It included 11 sociodemographic questions referring to the job position, 22 about protective or risk factors for sleep quality and the consequences of poor sleep quality, and 10 about the consumption of related medications/substances. As for the work modalities, 2 types of shifts were considered, namely shifts of 7-7-10 h (morning 8:00 am–3:00 pm, afternoon 3:00 pm–10:00 pm, and night 10:00 pm–8:00 am) and shifts of 12 h.

Sleep quality and its consequences were assessed using three validated scales with good psychometric properties (Table 1). The Insomnia Severity Index (ISI) [17,18,19] was used to evaluate sleep quality. The scale has a total score range of 0–28, where 0–7 indicates the absence of clinical insomnia, 8–14 indicates subclinical insomnia, 15–21 indicates moderate clinical insomnia, and 22–28 indicates severe clinical insomnia. The Patient Health Questionnaire-4 (PHQ-4) [20,21] is a self-administrated tool used for evaluating mental health in the population. It consists of the PHQ-2 [22,23], a shortened version of the PHQ-9 [24] for depression, and the GAD-2 [25], derived from the GAD-7 [26] for anxiety. By combining these two components, the PHQ-4 provides a concise four-item measure that assesses both depression and anxiety.

At the end of the questionnaire, an option was enabled for participants with scores greater than 7 points on the ISI (corresponding to at least subclinical insomnia) to provide an email address or telephone number so that the Occupational Medicine Service could contact them to offer strategies for treating sleep problems. Although anonymity was broken, this circumstance was recorded in the protocol.

Participants were asked whether they had received a formal diagnosis of any pathology from a qualified healthcare professional.

In this study, missing data were handled by excluding participants from specific analyses if they lacked data on the variable(s) of interest. Consequently, the sample size differs across analyses. Prior to conducting the main analyses, we performed preliminary checks for patterns in missing responses; no systematic or non-random patterns were detected, suggesting that the missing data mechanism did not introduce bias in the results.

A descriptive analysis of the data was performed. Frequencies, percentages, means with their standard deviation (SD), and Mann–Whitney U, Kruskal–Wallis K, Spearman rho, and Chi-Square tests were used. A confidence level of 5% was established. Data were analyzed using SPSS, Version 28.0 [28].

This study was performed in accordance with the Declaration of Helsinki on medical research in human beings in its latest version and with the applicable regulations on Good Clinical Practice. The confidentiality of the participants’ personal data was preserved in accordance with Organic Law 3/2018, of December 5, on the Protection of Personal Data and guarantee of digital rights. Informed consent was obtained from each participant. This study was approved on 24 October 2022 by the local bioethics committee of CAUSA together with the Ethics Committee for Drug Research of the Salamanca Health Area (code PI 2002101152).

## 3. Results

### 3.1. Sample

A total of 1121 questionnaires were completed, representing around 30.6% of all HCWs (see the calculation of the sample size in the supplementary files). In total, 61 percent of the questionnaires were answered completely and 39% partially. The characteristics of the study sample are described in Table 2.

Of the respondents, 26.4% (n = 297) filled out the telephone and/or email fields to be contacted by the Occupational Medicine Service.

### 3.2. Sleep Characteristics

**Figure 1:**
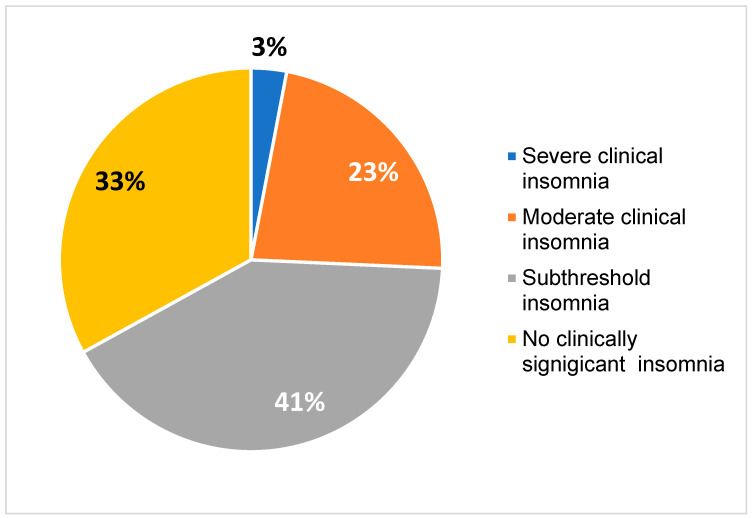
Pie chart of Insomnia Severity Index scores obtained of the sample during the study (n = 915).

The mean score on the ISI scale (n = 915) was 10.51 ± 5.8, corresponding to subclinical insomnia. Moderate clinical insomnia was present in 22.7% (n = 208) and severe clinical insomnia in 3% (n = 27) (Figure 1). In a total of 24 (3.1%) participants, the characteristics of a severe sleep disorder were detected.

Poorer sleep quality was observed in women, who obtained an average ISI score of 10.74 ± 0.2 (compared to 9.69 ± 0.4 for men), although the differences were not statistically significant (*p* = 0.325). ISI scores were positively correlated with age (correlation coefficient, r = 0.086; *p* = 0.010).

### 3.3. Interaction Between Work and Sleep

**Figure 2:**
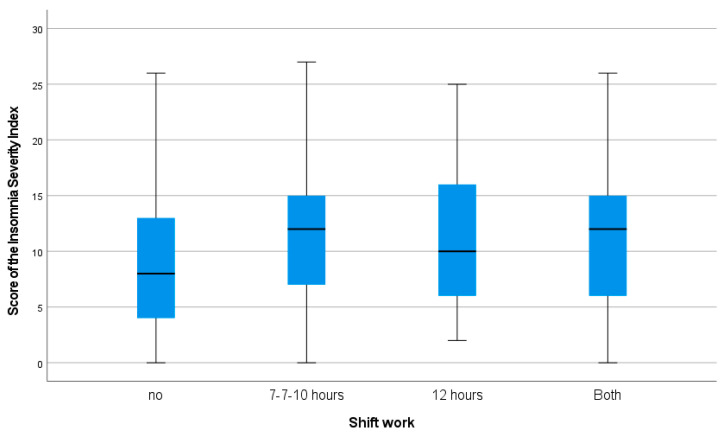
Boxplot of Insomnia Severity Index scores obtained during the study according to work schedule (n = 915).

Participants who worked shifts had poorer sleep quality than those on a continuous schedule. Specifically, the 7-7-10 h shift group had a mean ISI of 11.3 ± 0.2 (*p* < 0.001 vs. continuous schedule), the 12-h shift group had 11.3 ± 0.9 (*p* = 0.016), and the group working both shifts had 10.93 ± 0.8 (*p* = 0.012), whereas participants on a continuous schedule had 8.8 ± 0.3 (Figure 2). The differences were significant between participants who did not work shifts and those who worked 7-7-10h shifts (*p* < 0.001). However, no significant differences were found between the rest of the groups.

Nine participants (1.2%) had missed work in the previous month owing to poor sleep quality (n = 766).

### 3.4. Interaction Between COVID-19 and Sleep

COVID-19 sequelae were reported by 13.0% (n = 100) of participants, and this was a determining factor in poor sleep quality (*p* = 0.003). The odds ratio showed that the probability of sleep disturbances was 3.1 times higher in participants with COVID-19 sequelae than in those without (mean ISI scores 13.89 ± 5.9 vs. 10.33 ± 5.7, respectively), which was statistically significant (*p* < 0.001). No significant differences in the ISI scores were found between those who worked or did not work during the first waves of the COVID-19 pandemic (*p* = 0.209).

### 3.5. Psychiatric Comorbidities

Of those who completed the mental health questionnaires (n = 799), 28.4% (n = 226) had major depressive disorder and 33% (n = 264) had generalized anxiety disorder.

Of these, 94 were currently undergoing psychological or psychiatric treatment. Concurrent cases of depression and generalized anxiety disorder were observed in 166 individuals (20.8%).

#### Medication Consumption

Among the participants, 15.5% and 23.3% reported taking medications at least once a month to treat depression/anxiety or to fall asleep, respectively. Of the latter, 27.6% (n = 51) did so without a medical prescription; 8 participants reported using illegal drugs (cannabinoids, n = 6; stimulants and depressants, n = 1; stimulants, depressants, and cannabis, n = 1).

## 4. Discussion

This real-world study showed that sleep disorders, mainly insomnia, are highly prevalent among HCWs (25.7% for severe and moderate insomnia) and that shift work and the sequelae of COVID-19 may act as aggravating factors. Consequently, a significant proportion of workers face sleep problems that can affect their work performance and personal well-being. Remarkably, only 3.1% of the participants were currently diagnosed with a sleep disorder, and more than 26% of the respondents agreed to be contacted by the Occupational Medicine Service for assistance to solve their sleep problems.

### 4.1. Interaction Between COVID-19 and Sleep

We confirm that the sleep of HCWs was particularly affected by the COVID-19 pandemic and that these individuals were exposed to more stressful circumstances than other professionals. A systematic review and meta-analysis estimated the prevalence of insomnia symptoms in HCWs to be nearly 40% during the COVID-19 pandemic [7]. A 2021 meta-analysis of COVID-19-related stress and psychiatric symptoms in nurses (93 studies encompassing 93,112 nurses) [29] showed that over one-third reported stress, sleep disorders, and increased anxiety symptoms. The pooled prevalence of insomnia was 43% [29]. Furthermore, in a Spanish cohort of HCWs analyzed in April 2020 (n = 1422), over half reported symptoms of post-traumatic stress and anxiety disorders, and nearly 50% had experienced symptoms of depression, with women and younger individuals showing a higher risk [30]. Being a woman and working 12 h or 24 h shifts were risk factors for anxiety and depression. In our study, poorer sleep quality was observed in women and older individuals, although the differences were not statistically significant.

The probability of insomnia was 3.1 times higher in HCWs with COVID-19 sequelae, this difference being significant. Major depressive and generalized anxiety disorders were present in 28.4% and 33% of the samples, respectively, consistent with the frequently reported coexistence of insomnia and anxiety/depression [31]. In Spain, a meta-analysis of more than 82,000 individuals showed a pooled prevalence of anxiety symptoms and depression of 20% and 22%, respectively, during the COVID-19 pandemic; that of insomnia was 57% [32]. In another meta-analysis of studies comprising 271 Spanish HCWs, 33% of those exposed to COVID-19 reported depressive symptoms, 42% reported anxiety symptoms, 40% reported acute stress, 42% reported insomnia, and 37% reported burnout [33]. At our hospital, the prevalence of psychiatric symptoms among HCWs was also specifically analyzed during the pandemic through self-report surveys conducted during both waves [8]. Insomnia and anxiety were common (71.8% and 57.1%, respectively), although the frequency of both decreased in the second survey.

### 4.2. Underdiagnoses

The mean score on the ISI scale of the population analyzed in the present study corresponded to subclinical insomnia. Despite moderate and severe clinical insomnia being present in more than 25% of the participants, in only 3.1% of the total population was the trait of a sleep disorder detected. This frequency seems low considering the negative impact of insomnia on productivity and cognitive functioning in the medical profession [4,34,35]. Self-perceived sleep disturbances should be the first step before seeking medical evaluation, and barriers among HCWs for not doing so should be analyzed. Ideally, workplace-based interventions promoting mental health should incorporate a multidisciplinary approach to insomnia to detect it in early phases.

### 4.3. Interaction Between Work and Sleep

Of the 31.6% of the participants who worked overtime, less than 22% considered that these extra hours were properly paid. Low professional recognition may be linked to burnout and related symptoms [36]. A study carried out on 240 family doctors based at 70 health centers in Madrid revealed higher rates of insomnia and poorer sleep quality in those with a higher degree of burnout (39.7% vs. 7.3%) [37]. This problem needs urgent attention, as the daytime consequences of insomnia can also influence decision-making and patient care. Lack of sleep decreases the ability to discern and reflect emotions, which can reduce the clinician’s ability to show empathy and engagement [34,38,39]. In addition, the incidence of errors tends to be higher among HCWs with poor mental health [40,41].

Regarding the pharmacological strategies against insomnia, 23.3% of the participants reported taking medications at least once a month to help them sleep, and more than a quarter did so without a medical prescription. These percentages appear to be higher than previously reported by Andrés-Olivera et al. [8] during the first two waves of the pandemic at our hospital. Most individuals did not take benzodiazepines (68.9%; n = 42), and no significant increase in substance use was found compared to pre-pandemic levels. According to these data, substance use by HCWs may have been aggravated after the COVID-19 pandemic. It should be noted that many of the conventional drugs used to treat insomnia (benzodiazepines, non-benzodiazepine receptor agonists, melatonin receptor agonists, and tricyclic antidepressants) present the potential for abuse and relevant adverse effects [42,43]. In contrast, new therapeutic options, such as the dual orexin receptor antagonists, have been shown to reduce excessive wakefulness and exert effects on daytime functioning [5,43,44]. Therefore, it is important to tailor the treatment of insomnia based on the socio-occupational circumstances of each individual to prevent any negative impact on both personal and professional aspects [45].

According to our findings, working shifts can be an aggravating factor for insomnia, as previously described [6,46,47]. Overtime and demanding work conditions with lengthy shifts have been described as stressful circumstances that affect resilience and mental health outcomes among HCWs [47], as well as physicians’ cognitive skills and performance [48]. Flexible scheduling could enhance the sleep of physicians who work night shifts, thereby reducing their levels of fatigue and improving patient care.

Our study is subject to a series of limitations. First, the single-center design may prevent the extrapolation of our findings to other centers. Moreover, studies based on surveys may be affected by selection bias owing to the different motivations of the participants for expressing their opinions. Due to the cross-sectional design, causal effects of shift work and COVID-19 sequelae on insomnia cannot be asserted. Pre-existing mental health conditions and workload variability may have acted as confounding variables. Additional limitations may have been the potential recall bias in self-reported sleep assessments, the lack of objective sleep measures (e.g., polysomnography, actigraphy), and the lack of longitudinal data to assess causality. However, these results, which are from a large sample representing a relevant part of our center’s HCWs, contribute to a field where real-world data in the work setting are scarce. They could serve as a basis for future research on the effect of strategies to manage insomnia on the work performance and daily functioning of HCWs.

## 5. Conclusions

Insomnia is common among HCWs and affects mental health and work performance. It could be hypothesized that the sequelae of COVID-19 and shift work could trigger or aggravate sleep disorders, exerting bidirectional effects with coexisting medical and mental disorders.

Workplace-based interventions at the organizational level promoting mental health and well-being among HCWs should incorporate a perspective on sleep problems and their consequences. Successful management requires a multidisciplinary approach, whereby specialists in neurology, psychiatry, psychology, and occupational health, among others, work on the design of joint protocols that address insomnia. Large-scale studies are needed to test multidisciplinary strategies in this setting. The physical and mental well-being of these professionals is important not only at the personal level but also to ensure that patients receive high-quality clinical care.

## Figures and Tables

**Table 1:** Scales included in the questionnaire.

Scale	Parameters	Psychometric Properties
Insomnia Severity Index (ISI) [8,17,18,19]	Impact on quality of life	α = 0.82. Correlation: 0.35 for insomnia; 0.56 for impact of insomnia; 0.50 for dissatisfaction with sleep.
Patient Health Questionnaire (PHQ-4) [20,21,27]	Depression and anxiety	α > 0.8; r = 0.2 with Study Short Form-20.
Patient Health Questionnaire (PHQ-2) [22,23,26]	Major depressive disorder	The optimal PHQ-2 cut-off score was 3, achieved with a sensitivity of 74.6% and specificity of 93.9%. Area under the curve: 0.92 (CI 0.91–0.93).
Generalized Anxiety Disorder Scale-2 Questionnaire (GAD-2) [26]	Generalized anxiety disorder	Both the GAD-7 scale and its 2 core items (GAD-2) performed well (area under the curve, 0.80 to 0.91) as screening tools for anxiety disorders.

**Table 2:** Characteristics of the study sample.

Characteristic	Values	IC95%
Age, years (mean ± SD)	44.59 ± 11.78	
Sex (n, %)		
Women	760 (78.3)	10.31–11.16
Men	211 (21.7)	8.81–10.57
Work modality (n, %)		
Bachelor’s degree	700 (72.2)	
Permanent contract	309 (31.9)	
Temporary contract	191 (19.7)	
Job category * (n, %)		
A2	304 (31.4)	
A1	198 (20.4)	
C1-C2	195 (20.1)	
Overtime in the previous month (n, %)	307 (31.6)	
Extra hours paid	67 (21.8)	
Shift work (n, %)	647 (57.7)	
7-7-10 h schedule	542 (83.7)	10.84–11.83
12 h schedule	45 (6.9)	9.51–13.15
Both (in the last month)	59 (9.2)	9.26–12.6
Including nights	394 (51.4)	
Rotating shift	364 (47.5)	
Work during COVID-19 (n, %)	598 (78)	
COVID-19 sequelae (n, %)	100 (13)	

* A2: nurses, occupational therapists, nutrition graduates, and speech therapists; A1: graduate specialists; C1–C2: senior technicians, auxiliary care technicians, and pharmacy technicians.

## Data Availability

The data presented in this study are available upon request from the corresponding author. The data are not publicly available due to privacy or ethical restrictions.

## Supplementary Materials

The following supporting information can be downloaded at: https://www.mdpi/article/10.3390/jcm14051663/s1, Appendix S1: Sleep Screening Assessment Questionnaire; Supplementary S1: Sample size and the minimun number of participants; Supplementary S2: poster that was put on the hospital walls.
