# Supplementary material for: The Relevance of Insomnia Among Healthcare Workers: A Post-Pandemic COVID-19 Analysis
Source: J Clin Med. 2025 Feb 28;14(5):1663. doi: 10.3390/jcm14051663 (PMC11900261; doi:10.3390/jcm14051663)
Supplement: Supplementary file 1 [file jcm-14-01663-s001.zip › Appendix S1.pdf]

COMPLEJO ASISTENCIAL UNIVERSITARIO DE SALAMANCA
Paseo de San Vicente, 58-182
37007 Salamanca

Sacyl
SANIDAD DE CASTILLA Y LEÓN

Annex I.

CAUSA Sleep Screening Assessment Questionnaire 2022

Note: The answer options that have the ends of 0 and 10 indicated refer to the fact that it is possible to select the intermediate options.

Sociodemographic

number	Instructions	Item Wording 1	Item 2 Wording	Answer Options
1			Indicate your age in years	[years]
2	This item conditions the i51 *I have hormonal disorders related to menopause.*		What is your biological sex?	(1) Male;<br>(2) Women
3			What gender do you identify with?	(1) I feel like a man;<br>(2) I feel like a woman;<br>(3) Other
4			What is your completed level of studies?	1. Primary<br>2. High school<br>3. Technical studies,<br>4. University degree/bachelor's degree,<br>5. Master<br>6. Doctorate
6		Putting together all the time he worked for CAUSA (even having discontinued contracts)	Please indicate your years of experience working at the CAUSA. In the case where you have worked for less than a year, mark ZERO.	[years]
7		Regarding the current contract	What type of position are you filling?	1. A1 and A2 management staff (directors, deputy directors)<br>2. Heads of service and unit A1 and A2 (Heads of Service, Section and Unit, supervisors, post complement).<br>3. Others C1 and C2 (group, team and personnel leaders, maintenance manager, technician coordinator, installation driver).<br>4. A1 health personnel (Specialist graduates).<br>5. A2 health personnel (nurses, occupational therapists, graduates in nutrition, speech therapists).<br>6. C1 and C2 health personnel (Senior Technician, Auxiliary Care Technician, Pharmacy Technician).<br>7. Management and service staff A1, A2, C1, C2 and OAP (Graduates, Engineers and Librarians; Technical Engineers. Industrial or similar degree).<br>8. Administrative Assistant, maintenance personnel, Porters, Operators, Assistants).
8		Regarding the current contract	Indicate the type of contract you have	1. Owner<br>2. Indefinite (interim-long-term position, secondment, etc.)<br>3. Temporary<br>4. Residente (MIR/PIR/FIR/QIR/BIR/EIR).
9		Regarding the current contract	In the last month, have you worked overtime?	(1) Yes;<br>(2) No
10		Regarding the current contract	In the last month, have you worked more hours than you are contractually entitled to?	(1) Yes;<br>(2) No
11	This item conditions the i68 *Have you been worried because your job was in danger due to the COVID-19 pandemic?*		Have you worked at CAUSA during the first waves of the COVID-19 pandemic?	(1) Yes;<br>(2) No

COMPLEJO ASISTENCIAL UNIVERSITARIO DE SALAMANCA
Paseo de San Vicente, 58-182
37007 Salamanca

Sacyl
SANIDAD DE CASTILLA Y LEÓN

ISI Insomnia Severity Index

https://doi.org/10.1016/j.sleep.2011.06.019

The sum of responses to ISI items (from 12 to 18) conditions item 70.

number	Item Wording 1	Item 2 Wording	Answer Options
12	Please indicate the SEVERITY of your current (e.g., within the last 2 weeks) sleep problem(s):	Difficulty falling asleep	(0) Nothing;<br>(1) Mild;<br>(2) Moderate;<br>(3) Severe;<br>(4) Very serious
13	Please indicate the SEVERITY of your current (e.g., within the last 2 weeks) sleep problem(s):	Difficulty staying asleep:	(0) Nothing;<br>(1) Mild;<br>(2) Moderate;<br>(3) Severe;<br>(4) Very serious
14	Please indicate the SEVERITY of your current (e.g., within the last 2 weeks) sleep problem(s):	Waking up too early	(0) Nothing;<br>(1) Mild;<br>(2) Moderate;<br>(3) Severe;<br>(4) Very serious
15		How **SATISFIED** are you with your dream today?	(0) Very satisfied;<br>(1) Satisfied;<br>(2) Neutral;<br>(3) Not very satisfied;<br>(4) Very dissatisfied
16		To what extent do you feel your sleep problem **INTERFERES** with your daily functioning (e.g., daytime fatigue, ability to perform daily tasks/work, concentration, memory, mood, etc.)?	(0) Nothing;<br>(1) A little;<br>(2) Something;<br>(3) A lot;<br>(4) A lot
17		To what extent do you think **OTHERS are aware** of your sleep problem because of what it affects your quality of life?	(0) Nothing;<br>(1) A little;<br>(2) Something;<br>(3) A lot;<br>(4) A lot
18		How **CONCERNED** are you about your current sleep problem?	(0) Nothing;<br>(1) A little;<br>(2) Something;<br>(3) A lot;<br>(4) A lot

COMPLEJO ASISTENCIAL UNIVERSITARIO DE SALAMANCA
Paseo de San Vicente, 58-182
37007 Salamanca

Insomnia Daytime Symptoms and Impacts Questionnaire (IDSIQ)

https://doi.org/10.1007/s40271-020-00474-z

number	Item Writing	Answer Options
19	To what extent have you felt **clear** today?	(0) Nothing clear<br>(10) Very clear
20	To what extent have you felt **worried** today?	(0) Not at all concerned<br>(10) Very concerned
21	How frustrated **have you been** by the lack of sleep today?	(0) Not at all frustrated<br>(10) Not at all frustrated
22	To what extent have you felt **irritable** today?	(0) Not irritable at all<br>(10) Very irritable
23	To what extent have you felt **stressed** today?	(0) Not stressed at all<br>(10) Very stressed
24	To what extent have you felt **energetic** today?	(0) Not at all energetic<br>(10) Very energetic
25	To what extent have you felt **sleepy** today?	(0) Not at all drowsy<br>(10) Very sleepy
26	To what extent have you felt **awake** today?	(0) Not awake at all<br>(10) Very awake
27	To what extent have you felt with renewed **strength** today?	(0) With the strength not at all renewed<br>(10) With very renewed strength
28	To what extent have you felt **physical fatigue** today?	(0) No physical fatigue<br>(10) A lot of physical fatigue
29	To what extent have you felt **mental fatigue** today?	(0) No mental fatigue<br>(10) A lot of mental fatigue
30	To what extent have you been **able to concentrate** today?	(0) I have been completely unable to concentrate<br>(10) I was able to concentrate very well
31	To what extent have you been **forgetful** today?	(0) Not forgetful at all<br>(10) Very forgetful
32	How much has **it cost you to do your daily activities** today (e.g., reading, cleaning, working, studying)?	(0) It didn't cost me anything<br>(10) It has cost me a lot

PHQ2, GAD2 (the union of the two results in PHQ-4), item 15 of PHQ15

- PHQ2 Kroenke, K., Spitzer, R., & Williams, J. (2003). The patient health questionnaire-2 of a two-item screener validity depression. Med Care, 41, 1284–1292.
- PHQ4 Kroenke, K., Spitzer, R. L., Williams, J. B. W., & Löwe, B. (2009). An ultra-brief screening scale for anxiety and depression: The PHQ-4. Psychosomatics, 50(6), 613–621. https://doi.org/10.1176/appi.psy.50.6.613
- GAD2 Williams, J. B. W., Monahan, P. O., Löwe, B., Kroenke, K., Spitzer, R. L., & Lö, B. (2007). Metabolic syndrome View project Frontiers in Psychiatry Research Topic on coronavirus and mental health View project Anxiety Disorders in Primary Care: Prevalence, Impairment, Comorbidity, and Detection. https://doi.org/10.7326/003-4819-146-5-200703060-00004
- PHQ15 Actas Esp Psiquiatr 2010; 38(6):345-57 https://www.researchgate.net/publication/49712122_Validation_of_the_Spanish_Version_of_the_PHQ-15_Questionnaire_for_the_evaluation_of_physical_symptoms_in_patients_with_depression_andor_anxiety_disorders_DEPRE-SOMA_study

number	Item Wording 1	Item 2 Wording	Answer Options
33	Over the past two weeks, how often have you felt discomfort from the following issues?	Little interest or pleasure in doing things.	(0) Not at all;<br>(1) Several days;<br>(2) More than half of the days;<br>(3) Almost every day
34	Over the past two weeks, how often have you felt discomfort from the following issues?	Feeling discouraged, depressed, or hopeless.	(0) Not at all;<br>(1) Several days;<br>(2) More than half of the days;<br>(3) Almost every day
35	Over the past two weeks, how often have you felt discomfort from the following issues?	Feeling nervous, anxious, or very tense.	(0) Not at all;<br>(1) Several days;<br>(2) More than half of the days;<br>(3) Almost every day
36	Over the past two weeks, how often have you felt discomfort from the following issues?	Not being able to stop worrying or not being able to control worry.	(0) Not at all;<br>(1) Several days;<br>(2) More than half of the days;<br>(3) Almost every day
37		Over the past 4 weeks, how much has it bothered you to have trouble sleeping?	(0) Not at all;<br>(1) Several days;<br>(2) More than half of the days;<br>(3) Almost every day

Substances

number	Item Instructions	Item Wording 1	Item 2 Wording	Answer Options 1
38		Smoking regularly	More than 1 cigarette a day	(1) Yes<br>(2) No
39		Weekly I take	Alcohol	1.- No<br>2.- I take 3 or less<br>3.- Volume between 3 and 6<br>4.- I take more than 6
40		The number of coffees/teas/energy drinks I usually drink each day is: (If it's double coffee, count by 2. If it is an energy drink [330ml] count by 5).		(1) 0<br>(2) 1<br>(3) 2<br>(4) 3<br>(5) 4<br>(6) 5<br>(7) 6<br>(8) More than 6
41	This item conditions the i42 *The medication I take to sleep has a medical prescription*	I take 1 time a month or more	Sleep medication	(1) Yes<br>(2) No
42	Only if you take sleep medication (if i41=1)	The medication I take to sleep is prescribed by a doctor		(1) Yes<br>(2) No
43	This item conditions the i44 *The medication I take for depression or anxiety has a medical prescription*	I take 1 time a month or more	Medication for depression or anxiety	(1) Yes<br>(2) No
44	Only if you take medication for depression or anxiety (if i43=1)	The medication I take for depression or anxiety is prescribed by a doctor		(1) Yes<br>(2) No
45		I take 1 time a month or more	Illegal substances STIMULANTS (cocaine, amphetamines...)	(1) Yes<br>(2) No
46		I take 1 time a month or more	Illegal DEPRESSANT substances (opiates or others).	(1) Yes<br>(2) No
47		I take 1 time a month or more	Illegal substances CANNABIS TYPE.	(1) Yes<br>(2) No

Protective or risk factors, quality of life and occupational consequences

number	Item Instructions	Item Wording 1	Item 2 Wording	Answer Options
48		I am currently undergoing psychological or psychiatric treatment		(1) Yes;<br>(2) No
49		I currently have sequelae from having had COVID disease		(1) Yes;<br>(2) No
50		I have been diagnosed with a sleeping sickness		(1) Yes;<br>(2) No
51	Only if sex = female (if i2 = 2)	I have hormonal disorders related to menopause.		(1) Yes;<br>(2) No;<br>(3) Not applicable
52		I currently work night shifts.		(1) Yes;<br>(2) No
53		I do guard duty		(1) Yes. I do physical presence shifts, but not localized;<br>(2) Yes, I do localized guards, but with a non-physical presence;<br>(3) Yes, I do both physical and localized on-call duty.<br>(4) I don't do guard duty.
54		I have rotating shifts that cause me frequent changes in the hours I spend sleeping.		(1) Yes;<br>(2) No
55	This item conditions the i57 and i58	Duermo la siesta.		(1) Yes;<br>(2) No
56	Only you duerme the siesta (si i56=1)	The frequency with which I take a nap in a week is:		1. 0<br>2. 1<br>3. 2<br>4. 3<br>5. 4<br>6. 5<br>7. 6<br>8. 7
57	Only you duerme the siesta (si i56=1)	How long does he take a nap on average?		(1) Less than 15 minutes;<br>(2) Between 15 and 30 minutes;<br>(3) Between 30 and 45 minutes;<br>(4) Between 45 and 60 minutes;<br>(5) Between one hour and one and a half hours;<br>(6) Between an hour and a half and two hours;<br>(7) More than 2 hours
58		In the last month and due to fatigue I have noticed that I make more mistakes in my daily tasks.		(0) Strongly agree;<br>(10) Strongly disagree
59		To perform my work I need high concentration and the use of higher capacities (attention, perception and memory).		(0) Strongly agree;<br>(10) Strongly disagree
60		In the course of my work, there are days of concentrated and sustained attention for long periods of time		(0) Strongly agree;<br>(10) Strongly disagree
61		I take enough breaks during my workday to break the monotony and disconnect to resume proper attention in my work		(0) Strongly agree;<br>(10) Strongly disagree
62		The lack of attention in my work has a direct consequence of serious consequences		(0) Strongly agree;<br>(10) Strongly disagree

number	Item Instructions	Item Wording 1	Item 2 Wording	Answer Options
63		Sleep negatively influences my quality of life		(0) Strongly agree;<br>(10) Strongly disagree
64		Due to poor sleep quality in the last month	I've missed work	(1) Yes;<br>(2) No
65		In the last month and due to the poor quality of sleep I have difficulty	Doing my job	(0) No difficulty;<br>(1) A little difficulty;<br>(2) A lot of difficulty;<br>(3) Extreme difficulty
66		In the last month and due to the poor quality of sleep I have difficulty	Perform household chores	(0) No difficulty;<br>(1) A little difficulty;<br>(2) A lot of difficulty;<br>(3) Extreme difficulty
67		In the last month and due to the poor quality of sleep I have difficulty	Relate to others	(0) No difficulty;<br>(1) A little difficulty;<br>(2) A lot of difficulty;<br>(3) Extreme difficulty
68	Only if you worked during first waves (if i11=1)	Have you been worried that your job was in jeopardy due to the COVID-19 pandemic?		(0) No worry;<br>(1) A little worry;<br>(2) A lot of concern;<br>(3) Extreme concern
69		In the last month and due to the poor quality of sleep I have felt that the attention that work demands of me is greater than what I can offer.		(0) Strongly agree;<br>(10) Strongly disagree.

Treatment, subsequent contact and final message

number	Item Instructions	Item Writing	Answer Options
70	This item is conditioned by i12 to i18.<br>It will only be shown to those who have obtained a clinical insomnia score (scores greater than 15).<br>*Only if ISI≥15 (sum of ISI test item scores, which are 12 to 18)*<br>Total score categories:<br>0–7 = No clinically significant insomnia<br>8–14 = Subthreshold insomnia<br>15–21 = Clinical insomnia (moderate severity)<br>22–28 = Clinical insomnia (severe)	You are told that you may have problems with sleep. Occupational Medicine has set up an operation to treat specific sleep problems. If you want to be treated for your sleep problem , **please provide your phone number and email.**	[OPEN RESPONSE]

Final message for all participants

Thank you very much for your participation. Below you can find information about sleep hygiene: https://www.cop.es/colegiados/PV00520/Como%20dormir%20mejor.pdf
