# Supplementary material for: The Relevance of Insomnia Among Healthcare Workers: A Post-Pandemic COVID-19 Analysis
Source: J Clin Med. 2025 Feb 28;14(5):1663. doi: 10.3390/jcm14051663 (PMC11900261; doi:10.3390/jcm14051663)
Supplement: Supplementary file 1 [file jcm-14-01663-s001.zip › Supplementary S1 sample.pdf]

Supplementary files

The sample size and the minimun number of participants

The sample size was determined by the following formula:

$$n = \frac{N \cdot Z_{\alpha}^{2} \cdot p \cdot q}{d^{2} \cdot (N-1) + Z_{\alpha}^{2} \cdot p \cdot q}$$

Where:

- N is the whole population
- $Z_{\alpha}^{2} = 1{,}96^{2}$
- p is the proportion of the variable with a fixed value of 0,05
- q is the complement of p to one, being $q = p - 1$
- d is the maximum allowable error (5%)

Given the total population of 6,193 individuals (according to Human Resources data) and the corresponding calculations, the minimum required sample size is 362 participants.
