# Supplementary material for: The Relevance of Insomnia Among Healthcare Workers: A Post-Pandemic COVID-19 Analysis
Source: J Clin Med. 2025 Feb 28;14(5):1663. doi: 10.3390/jcm14051663 (PMC11900261; doi:10.3390/jcm14051663)
Supplement: Supplementary file 1 [file jcm-14-01663-s001.zip › Supplementary S2 poster.pdf]

Poster

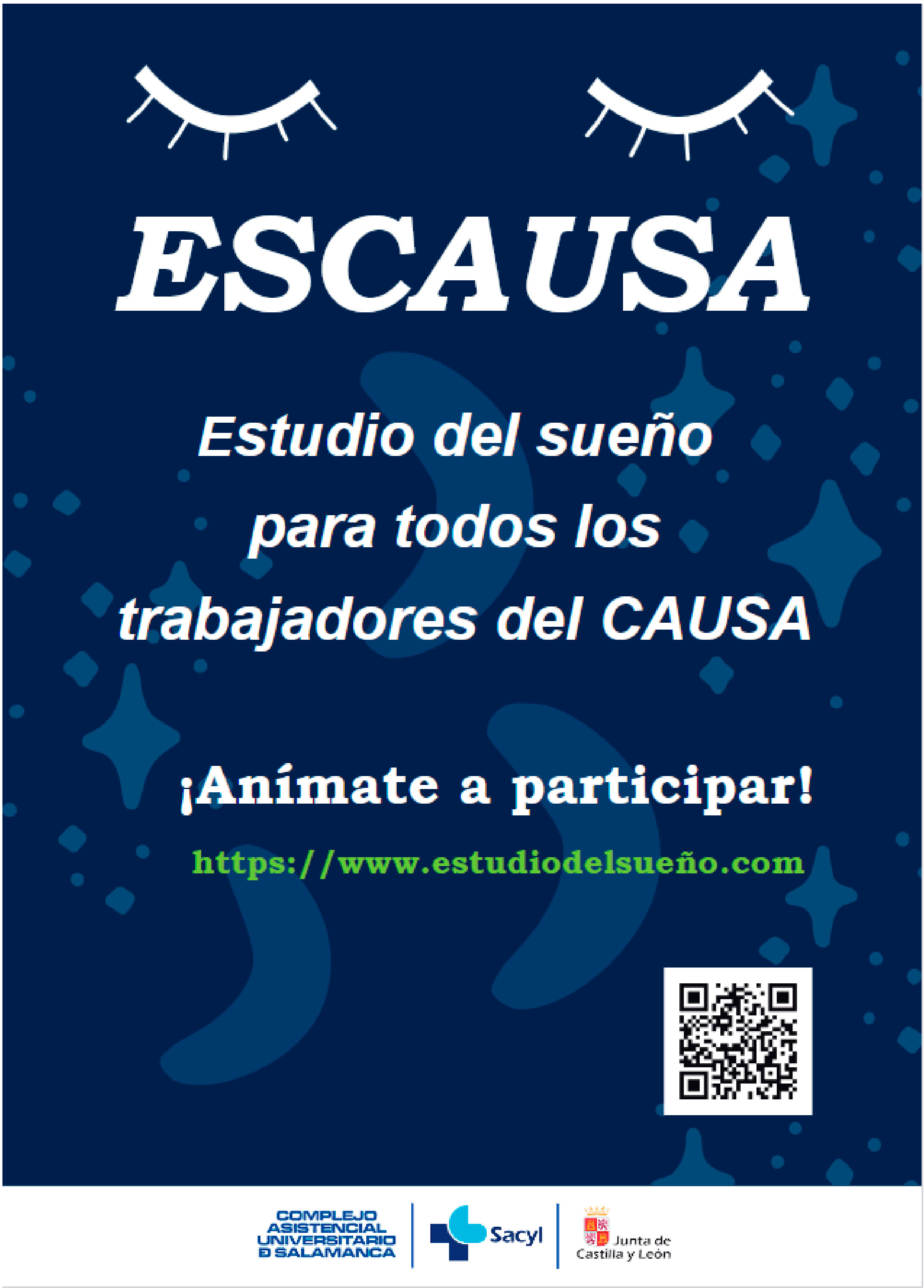
ESCAUSA
Estudio del sueño para todos los trabajadores del CAUSA
¡Anímate a participar!
https://www.estudiodelsueño.com
COMPLEJO ASISTENCIAL UNIVERSITARIO D SALAMANCA
Sacyl
Junta de Castilla y León
